# Case report: Brief, intensive EMDR therapy for borderline personality disorder: results of two case studies with one year follow-up

**DOI:** 10.3389/fpsyt.2023.1283145

**Published:** 2023-12-15

**Authors:** Laurian Hafkemeijer, Karin Slotema, Nicole de Haard, Ad de Jongh

**Affiliations:** ^1^Department of Adult Psychiatry, GGZ Delfland, Delft, Netherlands; ^2^Department of Personality Disorders, Parnassia Psychiatric Institute, The Hague, Netherlands; ^3^Academic Centre for Dentistry Amsterdam (ACTA), University of Amsterdam and VU University Amsterdam, Amsterdam, Netherlands; ^4^Research Department PSYTREC, Bilthoven, Netherlands; ^5^School of Health Sciences, Salford University, Manchester, United Kingdom; ^6^Institute of Health and Society, University of Worcester, Worcester, United Kingdom; ^7^School of Psychology, Queen’s University, Belfast, Ireland

**Keywords:** EMDR, borderline personality disorder, trauma, neglect, case studies, abuse

## Abstract

**Background:**

Exposure to adverse childhood events plays an important role in the development of borderline personality disorder (BPD). Emerging evidence suggests that trauma-focused therapy using eye movement desensitization and reprocessing (EMDR) can be beneficial for patients with BPD symptoms. To date, the effects of brief, intensive EMDR treatment for this target group have not been investigated in this population.

**Objective:**

This study aimed to evaluate the effects of a brief and intensive trauma-focused therapy course using EMDR therapy in two patients diagnosed with BPD who did not fulfill the diagnostic criteria for post-traumatic stress disorder (PTSD). It was hypothesized that this approach would be associated with a decline in the core symptoms of BPD, and that this would have an enduring long-term effect on patients’ diagnostic status.

**Method:**

Ten sessions of EMDR therapy were carried out across four consecutive treatment days, with the aim of processing patients’ core adverse childhood experiences. Both A-criterion-worthy memories (without intrusive reliving) and non-A-criterion-worthy memories that were considered responsible for the patients’ most prominent symptoms were targeted. The effects of EMDR therapy on trauma symptom severity and BPD diagnostic status (as established by the Structured Clinical Interview DSM-5) were determined. Additionally, the effects on psychological distress, quality of life, and difficulties in emotion regulation were determined at intake, post-treatment, and at 3-, 6-, and 12-months follow-up.

**Results:**

Both patients showed a strong decline in psychological distress and difficulties in emotion regulation, and reported an improvement in their quality of life. At post-treatment, and at 3-, 6-, and 12-months follow-up they no longer met the DSM-5 criteria for BPD.

**Conclusion:**

The findings of this small case study are in line with mounting evidence that a brief track of intensive trauma-focused therapy can result in long-term remission in patients with BPD. EMDR therapy seems to be a promising treatment approach for patients with BPD; however, the results need to be replicated in clinical trials.

## 1 Introduction

Borderline personality disorder (BPD) is characterized by a pattern of problematic interpersonal relationships, instability in affect regulation and impulse control, and recurring suicidal tendencies (1). International studies have shown that individuals fulfilling the diagnostic criteria for BPD display a high burden of disease and comorbidity (2), which is associated with reduced quality of life (3). The currently recommended first-line treatment options for BPD have proven to be extensive and costly (4, 5) and show intensive use of mental health services (6, 7). Effective interventions with a shorter duration are therefore a priority for this patient group.

Although post-traumatic stress disorder (PTSD) and BPD are classified differently, these mental health conditions often prove to be comorbid (8). Evidence suggests that 25–30% of individuals meeting the diagnostic criteria for PTSD also meet the diagnostic criteria for BPD (9). Conversely, at some point in their lives, 30–70% of individuals with a BPD diagnosis fulfill the diagnostic criteria for PTSD (9, 10). More importantly, there is a strong similarity between the symptoms considered characteristic of BPD and the symptom clusters of what is termed Complex PTSD [i.e., emotional regulation difficulties, disturbances in relational capacities, and the presence of negative beliefs; (11)], a mental health condition that has been found to be responsive to trauma-focused treatment (12, 13).

However, to be classified with PTSD exposure to an A-criterion-worthy event is necessary, but memories of other types of adverse childhood events also seem to play an important role in the development of BPD. For example, one study showed that 97% of individuals diagnosed with BPD were exposed to at least one type of childhood trauma, including abuse and neglect [unpublished data from (14)]. Also, Porter et al. (15) found that both emotional abuse (OR = 38.1) and neglect (OR = 17.7) were strongly associated with the presence of BPD.

Despite the strong association between exposure to childhood adverse events and symptom clusters that are considered characteristic of BPD (16, 17), childhood adverse events have not yet been the primary target for BPD treatment. However, processing childhood memories could be a promising treatment approach for these individuals. The adaptive information processing (AIP) model, which is the theoretical framework of EMDR therapy, clarifies the impact of traumatic experiences on functioning and provides a rationale for utilizing EMDR in the treatment of BPD (18). This model is based on the premise that many forms of psychopathology, with PTSD being the most salient example, are the result of disruptive experiences (in the form of fearful images, dysfunctional cognitions, negative emotions, and physical sensations) that have occurred since the time of the event. For example, patterns of childhood maltreatment have been found to predict problems in emotion processing and regulation in emerging adulthood (19). Furthermore, trauma-focused psychotherapy has been proven to positively influence emotion regulation difficulties in individuals with severe PTSD who have been exposed to early childhood trauma (20). Given that emotion regulation problems are a core symptom of BPD, it seems reasonable that trauma-focused treatment would be a promising therapy for this patient group.

Indeed, evidence indicates that trauma-focused psychotherapies are effective in the treatment of PDs and BPD (21–24). To date, few studies have evaluated the impact of trauma-focused treatment on BPD symptoms and have demonstrated a significant decrease in borderline symptoms (25). Importantly, no increase in self-injurious behaviors, suicide attempts, or hospitalization was noted, whereas the mean weighted dropout rate during the PTSD treatment was low (17%). To this end, in particular EMDR therapy has proven to be a useful and effective treatment for patients with PD who do not fulfill the diagnostic criteria for PTSD (21, 22, 26). A randomized controlled study of 97 outpatients with PD as the main diagnosis, showed that psychological distress and personality dysfunction decreased significantly after only five sessions of EMDR therapy compared to wait list (22). Importantly, EMDR therapy proved to be not only effective for the debilitating effects of exposure to A-criterion-worthy events but also for the treatment of memories related to emotional abuse, neglect, and other distressing life events, such as divorce or severe physical illness (27).

In recent years, also brief and intensive trauma-focused treatment has been found to be a feasible and safe treatment approach for individuals with clinically elevated symptoms of BPD [e.g., (28)]. For example, an uncontrolled outcome study among 45 patients diagnosed with both PTSD and BPD (23) found that BPD symptom severity decreased from pre- to post-treatment, and at 12-month follow-up, and 73% of the patients no longer met the criteria for BPD according to the SCID-5-P (Structured Clinical Interview for DSM-5) The 8-day treatment in this study consisted of a combination of Prolonged Exposure and EMDR therapy in an inpatient treatment setting. To date, intensive treatment with only EMDR therapy has not been explored in patients with BPD so far.

Therefore, in the present pilot study we offered 10 sessions of EMDR therapy lasting 90 min within four consecutive days to two people diagnosed with BPD, not fulfilling the diagnostic criteria for PTSD. Based on the findings of previous studies (22, 23, 26, 28), we hypothesized that BPD symptoms would significantly decrease using an intensive track of EMDR therapy. In addition, we were interested in the extent to which the treatment would affect psychological distress, difficulties in emotion regulation, quality of life and patients’ diagnostic status 1 year after the termination of therapy.

## 2 Methods

### 2.1 Procedures

Both patients underwent an intake session in which the Clinician Administered PTSD Scale for DSM-5 [CAPS-5; (29)] was administered to exclude a diagnosis of PTSD, whereas the SCID-5-P was used to determine the presence of PD. Although many of their symptoms seemed to be the result of traumatic experiences, neither fulfilled the diagnostic criteria for PTSD (30).

After being informed about EMDR therapy both patients gave their permission for participation and signed an informed consent form.

### 2.2 Measures

#### 2.2.1 Clinician-administered PTSD scale for DSM-5 (CAPS-5)

A Dutch translation of the CAPS-5 was used to measure the severity of PTSD symptoms (31). The CAPS-5 is a structured diagnostic instrument consisting of 20 questions. The severity of PTSD symptoms was scored on a scale of 0 to 4 (absent, mild, moderate, severe, and extreme, respectively), and the 20 questions together resulted in a total severity score. The other ten questions concerned the duration of PTSD symptoms, dissociative symptoms, and the negative effects of PTSD symptoms on different life domains (e.g., social contact and work/school) (32). The total CAPS-5 score demonstrates high internal consistency (α = 0.90) (29). All measurement moments are shown in Table 1.

**TABLE 1:** Administration schedule of the questionnaires.

	Baseline	Day 1	Day 2	Day 3	Day 4	3 month follow up	6 month follow up	12 month follow up
SCID-P-5*	x					x	x	x
CAPS*	x					x	x	x
OQ-45*		x	x	x	X	x	x	x
DERS*		x			X	x	x	x
MHQoL*		x			X	x	x	x
CTQ*		x						
LEC*		x						

*SCID-P-5, structured clinical interview for DSM-5 personality disorder; CAPS, clinician-administered PTSD scale for DSM-5, OQ-45: outcome questionnaire-45; DERS, difficulties in emotion regulation skills; MHQoL, mental health quality of life; CTQ, childhood trauma questionnaire; LEC, life events checklist for DSM-5.

#### 2.2.2 Structured clinical interview for DSM-5 personality disorders (SCID-5-P)

The SCID-P-5 is a structured clinical diagnostic interview used to determine whether someone meets the criteria for a personality disorder (PD) according to the DSM-5 (30). The Dutch translation of the SCID-P-5 contains 135 questions that are rated on a 3-point scale. Data on the reliability and validity of the Dutch SCID-5-P are not yet available, but they are expected to be equal to the previous version, which is the Structured Clinical Interview for DSM-IV Axis II Personality Disorders (SCID-II) (33). All measurement moments are shown in Table 1.

#### 2.2.3 Childhood trauma questionnaire—Short form (CTQ-SF)

The CTQ-SF is a self-report questionnaire intended as a screening tool to detect maltreatment during childhood in both clinical and non-referred groups (34, 35) and consists of five subscales. The subscales are physical and emotional neglect and physical, emotional, and sexual abuse. Respondents were asked to give a rating of between 1 and 5 on a five-point Likert scale for 25 statements about childhood trauma. This questionnaire also measures the severity of the exposure to childhood maltreatment (none, moderate, severe and extreme). The Dutch version of this questionnaire will be used with scales with good to excellent internal consistency (α = 0.89–0.95). Only the physical neglect scale has questionable internal consistency [α = 0.63; (35)].

#### 2.2.4 Life events checklist for the DSM-5 (LEC-5)

The Dutch translation of the LEC-5 has been used (32). This self-report questionnaire estimates exposure to potentially traumatic life-events (36). The LEC-5 distinguishes 17 potentially traumatic experiences (e.g., physical violence and natural disasters) as well as the type of exposure to a potentially traumatic event [1 = it happened to me, 2 = I witnessed it, 3 = I have taken note of it, 4 = in the context of work, 5 = I am not sure, 6 = does not apply to me; (31)]. The LEC was administered at baseline. Although the Dutch version has not yet been evaluated, agreement for the original scale is substantial (κ = 0.61) (36).

#### 2.2.5 Mental health quality of life (MHQoL) questionnaire

The MHQoL is a standardized, self-report questionnaire that measures quality of life. This questionnaire was developed for people with mental health problems (37) and consists of eight questions. The first seven questions represent seven different domains of mental health (e.g., self-image, relationships, and mood). Patients rated their degree of satisfaction with these domains on a four-point Likert Scale. The seven questions combined gave a total score. On the eight questions, the patients were asked to fill out a visual analog scale about their general psychological wellbeing (0, the worst you can imagine; 10, the best you can imagine). The MHQoL was administered on days 1 (pre-treatment), 4 (post-treatment), at 3-month follow-up, at 6-month follow-up and 12-month follow-up. Internal consistency of the Dutch version has proven to be high for the total score (α = 0.85) (37). All measurement moments are shown in Table 1.

#### 2.2.6 Difficulties in emotion regulation scale (DERS)

The DERS is a self-report questionnaire that measures difficulties in emotion regulation (38). Respondents are requested to indicate the frequency of 36 emotion regulation statements on a five-point Likert scale. The questionnaire consists of six subscales: lack of emotional clarity, lack of emotional awareness, impulsivity, non-acceptance of emotional responses, limited access to emotion regulation strategies and difficulties engaging in goal-directed behavior. Whereas the English DERS has demonstrated excellent internal consistency in a BPD sample (*a* = 0.94) (38), the subscales of the Dutch version have shown good internal consistencies in an adolescent sample [average *a* = 0.81; (39)]. All measurement moments are shown in Table 1.

#### 2.2.7 Outcome questionnaire (OQ-45)

The OQ-45 is a self-report questionnaire (40) that measures different domains of psychosocial functioning, such as symptom distress, interpersonal relations and social role (41). The Dutch version of the OQ-45 contains 45 items that are scored on a five-point Likert scale (never, rarely, sometimes, often, almost, and always). The OQ-45 has demonstrated excellent internal consistency for the total score in clinical samples (α = 0.93), but questionable internal consistency for the specific social role subscale (α = 0.69) (41). All measurement moments are shown in Table 1.

### 2.3 Treatment

Eye movement desensitization and reprocessing therapy is a standardized eight-phased trauma-focused therapy. It consists of dosed attention directed at a disturbing memory while simultaneously engaging in another concurrent (dual attention) task (18, 42). The EMDR therapy in this study was performed according to the guidelines of Shapiro (18) and the Dutch version of the EMDR standard protocol (43). In the present study EMDR was applied in that patients were requested to imagine the most disturbing part of a traumatic event while taxing their working memory capacity by visually following the therapist’s finger movements or other working memory-demanding elements to maximize working memory load (44). The EMDR therapists were experienced therapists who were certified as an EMDR Europe practitioner (LM) or trainer (AdJ) according to the guidelines of the EMDR Europe Association (EMDREA).

### 2.4 Participants

Two patients diagnosed with BPD were recruited through a call on the world’s largest professional network on the Internet, “LinkedIn.” Patients could participate if they had BPD as a primary diagnosis according to the DSM-5 criteria and the ability to speak and understand Dutch or English. Exclusion criteria were the presence of a PTSD diagnosis and *acute* current suicidal intention. The first two people who volunteered and fulfilled the criteria for inclusion were enrolled as study participants. The names mentioned in this case study are fictitious. In addition, essential background and personal details of both patients were changed to ensure anonymity and make recognition impossible.

#### 2.4.1 Amy

Amy, a single woman of 42 years old, reported emotional neglect with multiple memories of situations in which she did not feel heard or seen and extreme emotional abuse, of which the latter had the greatest impact on her daily life. Table 2 shows the results of the Life Events Checklist for the DSM-V (LEC-5), which provides a representation of Amy’s adverse life events. For example, she had witnessed several events of physical violence and had been exposed to multiple accidents; such as a pressure cooker exploding on her face and her hair catching fire when she was young.

**TABLE 2:** Life Events Checklist for DSM-5 (LEC-5).

	Fire or explosion	Transportation accident	Assault with a weapon	Sexual assault	Severe human suffering	Physical assault	Combat or exposure to a war-zone	Captivity	Any other very stressful event or experience
Amy	x	x	X	x	x				
Kate		x		x		x	x	x	x

On admission, Amy was not in therapy; however, she reported an extensive treatment history, including different psychotherapies and hospitalizations. She suffered from suspicious thoughts and feelings, and often felt that she was not good enough. In romantic relationships and friendships, she felt worthless. Furthermore, she tended to be submissive to others in their relationships. Amy met the following criteria for BPD according to the SCID-5-P; a pattern of instability in interpersonal relationships, frantic effort to avoid abandonment, persistently unstable self-image, affect lability, chronic feelings of emptiness and paranoid ideas or dissociative ideations caused by stress.

#### 2.4.2 Kate

Kate is a 31-year-old woman, born in Somalia during war. She grew up in an unsafe family situation including physical and verbal violence. Table 2 provides an overview of Kate’s adverse life events. She reported multiple events that met the A-criteria for PTSD (30). During childhood, she was sexually and physically abused by a nephew, and as an adult she was exposed to sexual abuse multiple times. Kate also had been the witness of many war crimes, and as a refugee she almost drowned. Despite these traumas she did not exhibit any PTSD symptoms. With the aid of the CAPS-5, it became clear that Kate did not experience any feelings when she talked about the horrible events she had been exposed to. She described two people: “Kate before the war and after the war.” She suffered the most from the physical abuse by her nephew when she was 7 years old. Furthermore, she had a history of extreme emotional abuse, physical abuse, sexual abuse, and emotional neglect during childhood.

Kate’s most prominent symptoms were a low self-esteem and fear of losing loved ones. She indicated that it was difficult for her to open up to someone, and emotionally connect with others. Based on the administration of the SCID-5-P, Kate met the following criteria for BPD: a pattern of instability in interpersonal relationships, frantic effort to avoid abandonment, impulsivity, persistently unstable self-image, affect lability, a chronic feeling of emptiness and an intense, inadequate feeling of anger that is hard to control. Kate indicated that she had had schema focused treatment for 3 years prior to admission into this study and completed a track of cognitive behavioral therapy.

### 2.5 Case conceptualization

In the first session, a case conceptualization was performed by two experienced EMDR therapists. This was based on a trauma-focused approach, followed by a symptom-focused approach. First, memories of traumatic events fulfilling the A criterion for PTSD were identified (intrusive memories prior to non-intrusive memories). All the memories were placed in a hierarchy based on their subjective units of disturbance (SUD). Second, the most pronounced and distressing symptoms were inventoried, after which the memories (of childhood adverse events) that gave rise to or worsened these symptoms were identified and ordered and structured along a timeline. Then, these memories were placed in a hierarchy based on their subjective units of disturbance (SUD). The case conceptualization was based on the principle that memories with the highest SUD would be treated first.

## 3 Results

### 3.1 Course of treatment

#### 3.1.1 Amy

Amy did not report any intrusive memories. However, several non-intrusive memories of A-criterion events were selected, such as a severe physical assault on her sister that she witnessed, and another violent incident. During the case conceptualization session, Amy mentioned experiencing distrust, self-sacrifice, and a negative self-image as her main symptoms. One specific memory that seemed to significantly influence her low self-esteem was an event in which her mother expressed that she had never wanted children. Most of Amy’s memories were related to emotional neglect. Notably, these memories evoked a high level of emotional distress and had high SUD ratings. She had not realized the extent of the impact these memories had on her before.

As Amy progressed through the desensitization phase and various memories were successfully processed, she began to understand more clearly the influence of traumatic events on her self-perception and worldview. Consequently, she began to believe that there was nothing inherently wrong with her. This new perspective has led to increased self-compassion and a reduction in self-criticism. Additionally, during the therapy sessions, she learned to view others, including her mother, with greater empathy. She gradually recognized the deficiencies her mother had experienced, which prevented her from receiving attention and care for Amy.

During the desensitization phase of the sessions, Amy noticed occasional suspicion toward the therapists. By allowing this feeling to be present in the session, acknowledging it briefly, and continuing with the EMDR therapy, the intensity of this feeling diminished. She realized that it was primarily a response to escalating tension, something she also recognized in her daily life. Cognitive interweaves (18), which are short, open-ended questions aimed at providing functional and supportive information during a session, proved to be helpful for Amy to gain a different perspective and take better care of herself. For example, when asked, “What would you like to do for that little girl now?” Amy would respond, “I would give her a hug and tell her that she matters.” The SUD scores decreased rapidly and the sessions consistently ended in a positive manner. Amy increasingly felt a sense of self-worth, and began to feel stronger.

The Flashforward technique (45) was used to prepare Amy for a future confrontation with her mother, a situation she had been avoiding and felt a great deal of anxiety about. Her catastrophic thought was that her mother would reject her. Once the overall disturbance related to this catastrophic fantasy significantly decreased, Amy became fully convinced that she could handle this confrontation and that it was necessary to break free from her avoidance behavior and establish a new, healthier relationship with her mother.

#### 3.1.2 Kate

Kate reported numerous memories that met the A-criterion for PTSD, including war crimes in Somalia, memories of being a victim of both physical and sexual abuse from a young age, and the situation where she nearly drowned while attempting to flee from Somalia. Initially, she was reluctant to face the latter situation and preferred to consider it a “funny scenario in a Hollywood film.” However, when the memory was intensified by asking Kate to create a mental image of the possibility of drowning and formulating the negative cognition “I am powerless,” she reported disturbance and a high SUD level associated with the image. During the sessions, she could feel and re-experience the danger of the situation, and expressed a desire to escape, but the cognitive interweave “Are you safe now?” helped her continue the therapy. Whereas Kate previously described herself as “a Kate before and a Kate after the war,” she increasingly realized that all the significant events had been part of who she was before EMDR treatment.

The memories related to her primary complaints were also addressed. One significant symptom cluster was Kate’s low self-esteem, which was identified during the case conceptualization session as having originated in her early childhood, where girls were considered less valuable than boys in her culture. Memories from various situations were processed. However, despite the high working memory load, Kate often blamed her younger self and found it difficult to view the events from a different perspective. For instance, she remained convinced that the rape she experienced at the age of seven was her own fault. Kate frequently experienced “looping” (this happens when a patient is stuck on negative thoughts or beliefs) during the EMDR therapy sessions. Cognitive interweaves were also helpful for Kate in accessing new information, enabling her to realize that the young girl was not to blame but should have been protected by others. This allowed her to look at herself with more compassion and feel anger toward the perpetrators. Kate’s negative self-image was further reinforced by memories of events that she was bullied as a child. These memories were also processed. Additionally, she gained insight into how hard she had always worked to seek approval from others and to compensate for her negative self-image. By the end of the treatment, Kate recognized herself primarily as “a fighter,” “brave,” and “worthy,” and she truly felt that she no longer needed to be ashamed of her past.

### 3.2 Change in psychological distress, quality of life, and emotion regulation difficulties

At the start of therapy both patients scored much higher than 56 on the OQ-45, the cutoff score between the normal and patient populations (46). During the 4 days of intensive EMDR therapy both patients reported a decrease in psychological distress and dysfunctioning as measured using the total score of the OQ-45 (see Table 3). Three months after the treatment, Amy reported a low level of psychological distress. The total Kate score increased from three to 12 months of follow-up.

**TABLE 3:** Course of psychological distress (OQ-45), difficulties in emotion regulation (DERS) and quality of life (MHQoL) over the measurement moments.

	DERS*					MHQoL*					OQ-45*						
	**Day 1**	**Day 4**	**3 month follow up**	**6 month follow up**	**12 month follow up**	**Day 1**	**Day 4**	**3 month follow up**	**6 month follow up**	**12 month follow up**	**Day 1**	**Day 2**	**Day**	**Day**	**3 month follow up**	**6 month follow up**	**12 month follow up**
													**3**	**4**			
Amy	136	53	52	51	45	16	19	17	18	20	81	45	37	23	22	19	23
Kate	127	79	78	75	96	6	17	15	13	15	120	85	77	39	64	62	81

*DERS, difficulties in emotion regulation skills; MHQoL, mental health quality of life; OQ-45, outcome questionnaire-45.

At baseline Amy reported an average score and Kate had a much lower quality of life as indexed by the MHQoL than the general population. From baseline to post-treatment, both patients showed an increase in their quality of life followed by a decrease. From the 6-month follow-up until the 12-month follow-up, both patients reported an increase in quality of life.

Both patients showed a decrease in difficulty with emotion regulation over time (Table 3). The greatest decrease in difficulty was observed during the trajectory of EMDR therapy. These improvements were maintained in all follow-up measurements.

### 3.3 Change in PTSD symptom severity and diagnostic status

At baseline, Amy showed symptoms of PTSD, but did not fulfill the diagnostic criteria for PTSD. At 3-month follow-up PTSD symptoms strongly decreased. Additionally, at six follow-up measurements, a further decline in these symptoms was observed. Amy indicated experiencing more peace in her life, as well as the opportunity “to do and see exactly what I am, and to really take control again, where I did not feel this way before.”

**FIGURE 1:**
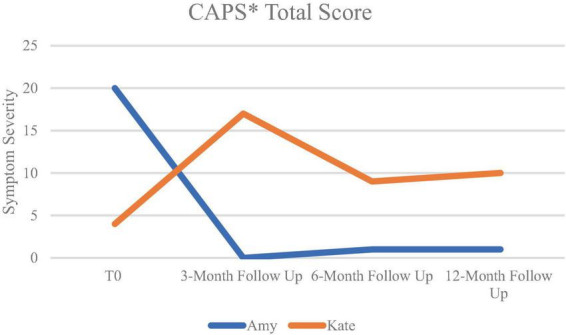
Means of CAPS-scores over time. *CAPS, clinician-administered PTSD Scale for DSM-5; T0: baseline measurement.

After EMDR therapy Amy no longer required further treatment. Kate reported no PTSD symptoms at baseline. However, at the 3-month follow-up, the patient fulfilled the diagnostic criteria for PTSD. After continuation of EMDR therapy by an independent psychologist, PTSD symptoms started to decrease again. At the 6-month follow-up, the patient no longer fulfilled the diagnostic criteria for PTSD. Figure 1 shows the mean CAPS scores at different measurement points. After the course of intensive EMDR therapy, Kate continued schema-focused therapy delivered by her psychologist. From post-treatment to the first follow-up measurement at 3 months she received six sessions of schema-focused therapy and three sessions of imaginary re-scripting. From the 3-months follow-up to the 6-months follow-up she received five sessions of schema-focused therapy and five sessions of EMDR therapy, and from the 6- to 12 months follow-up this treatment was continued.

### 3.4 Change in BPD symptom severity and diagnostic status

**FIGURE 2:**
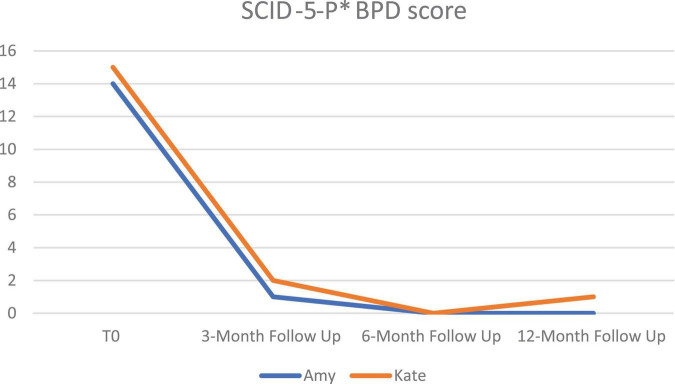
Mean SCID-5-P scores of BPD over time at pre-treatment (T0), 3-month follow-up, 6-month follow-up and 12-month follow-up. *BPD, borderline personality disorder; SCID-5-P, structured clinical interview for DSM-5; *SCID-5-P, structured clinical interview For DSM-5 personality disorder.

At post-treatment, and at all follow-up measurement moments, neither of the patients met the DSM-5 criteria of BPD according to the SCID-5-P (see Figure 2).

## 4 Discussion

Longitudinal studies have demonstrated the tremendous impact of adverse childhood experiences on the development of a wide variety of mental health conditions, including PDs (47, 48). However, to date, only a few studies have evaluated the effects of trauma-focused treatment on the symptoms of mental health conditions. In this study, two patients received 10 sessions of EMDR therapy for four consecutive days, focused on reprocessing the memories seemingly involved in the persistence of their main symptoms of their pathology. The results showed a strong decline in psychological distress, difficulties in emotion regulation, and improvement in the patients’ quality of life. At the 3-, 6-, and 12-months follow-up measurements neither patient fulfilled the diagnostic criteria for BPD.

The results of the present study are consistent with those of earlier case studies of patients diagnosed with BPD (21, 26). The results are also in line with two uncontrolled studies among patients with PTSD and comorbid BPD (23, 49), and a study among individuals diagnosed with BPD without the presence of PTSD (22). Furthermore, consistent with two previous studies, the effects on BPD symptoms (28), as well as BPD diagnostic status (23), could be maintained 1 year after therapy.

Remarkably, until now, almost all studies on the treatment of individuals with BPD included patients who were also classified as having PTSD (25). However, the majority of individuals with a PD do not meet the diagnostic criteria for PTSD, because the adverse childhood events they had been exposed to do not meet the A-criterion of the PTSD classification in accordance with the DSM-5, or they do not report sensory-based intrusive images of these events (30, 50, 51), such as Kate and Amy. To this end, it is important to note that EMDR therapy has been found to be also effective in patients with a PD focused on non-A-criteria worthy memories, such as those involving emotional abuse, neglect, and other distressing life events (27) similar to the two patients in the present case study. Interestingly, one of the two patients (Kate) no longer met the diagnostic criteria for BPD 3 months after treatment, but unexpectedly met those for PTSD. One possible explanation for this could be that Kate acquired strong survival strategies to regulate her emotions growing up in the war. Probably because of this, it proved difficult for Kate to allow her to experience emotions when the therapist asked her about the memory of fleeing the war. Kate was, in fact, a person with an overregulated affect, who was not easily overwhelmed with tears, who avoided feelings, and who was well capable to suppress and avoid emerging re-experiences. It is quite possible that this form (“overregulation of distress”) was the reason why she did not fulfill the criteria for PTSD at baseline (11). In short, it is conceivable that Kate’s treatment led to a reversal of the inhibition of previously overregulated emotions that gradually made the memories more accessible. The second treatment with EMDR therapy resulted in PTSD remission.

This study has several limitations. First, like any case study, it offers little basis for generalizing results to other clinical groups and contexts or for making predictions about future developments. Additionally, recruitment through social media could have attracted patients with mild problems; however, the questionnaire scores were within the clinical range. Conversely, to the best of our knowledge, this is the first case study to examine the effects of brief, intensive trauma-focused treatment using EMDR therapy in patients with BPD without PTSD. Only one earlier study (52) reported the effects of 20 weekly sessions of therapy in a 33-year-old female with BPD. She showed improvement in symptoms of borderline personality disorder, dissociative symptoms, depression and anxiety symptoms, which were maintained for 3 months after treatment. However, in this study a phase-based treatment approach was used, which started with a stabilizing phase using Resource Development and Installation (RDI) that lasted four sessions, followed by a trauma processing phase, and a “personality rehabilitation phase.” In our study the therapists immediately started with trauma processing in the first treatment session, treating the most disturbing memory first. Second, both self-report measurements and clinical interviews at several measurement points (at baseline, 3-, 6-, and 12-month follow-ups) were conducted, providing both a subjective and objective evaluation of the long-term therapeutic effects. Third, given the promising results, this study adds to the support of Shapiro’s Adaptive Information Processing (AIP) model, thereby underlining the importance of a trauma-focused approach to the treatment of patients with BPD and providing hope for further improvement of treatment outcomes for this and other diagnostic groups.

In conclusion, the findings of these case studies support the notion that reprocessing meaningful memories that are believed to underlie patients’ present symptoms within a brief time period of only 4 days not only improves PTSD symptoms but also core symptoms of BPD and may even result in long-lasting remission of BPD. Thus, replication in larger samples and clinical trials is required. To this end, we are currently awaiting the results of a randomized controlled outcome study on the effectiveness of EMDR therapy in a large group of people with a wide range of personality disorders (53), with and without PTSD.

## Data availability statement

The original contributions presented in this study are included in the article/supplementary material, further inquiries can be directed to the corresponding author.

## Ethics statement

Ethical approval was not required for the studies involving humans because the treatment was performed in accordance with the regulations for research as stated in the Declaration of Helsinki and the Dutch Medical Research on Humans Act (54) concerning scientific research. The measures that were used were standard routine outcome measurements and the same as those of our TEMPO study [(53); approved by the Medical Ethics Committee nr MEC-2020-0583]. The studies were conducted in accordance with the local legislation and institutional requirements. The participants provided their written informed consent to participate in this study. Written informed consent was obtained from the individual(s) for the publication of any potentially identifiable images or data included in this article.

## Author contributions

LH: Conceptualization, Data curation, Investigation, Project administration, Visualization, Writing – original draft, Writing – review and editing. KS: Writing – review and editing. AdJ: Conceptualization, Project administration, Writing – original draft, Writing – review and editing. NdH: Visualization, Writing – review and editing.
